# Assessment of the effect of health education on mothers in Al Maki area, Gezira state, to improve homecare for children under five with diarrhea

**DOI:** 10.4103/1319-1683.74332

**Published:** 2010

**Authors:** Huda M. Haroun, Mohamed S. Mahfouz, Mohamed El Mukhtar, Amani Salah

**Affiliations:** *Faculty of Medicine, University of Gezira, Sudan*; 1 *Department of Family and Community Medicine, Jazan University, Kingdom of Saudi Arabia*; 2 *Ministry of Health, Sudan*

**Keywords:** Diarrhea disease, home management and health education

## Abstract

**Introduction:**

Home care of under-five children is one of the most important interventions in the control of diarrheal diseases. It has a significant impact in reducing childhood mortality and morbidity.

**Objective:**

This study was conducted to evaluate the effect of health education on home care of under- five children with diarrheal disease.

**Materials and Methods:**

This is a quasi- experimental study, held in Al Maki neighborhood, which is located in Greater Wad Medani locality, Gezira State, Central Sudan. The study targeted a random sample of 118 mothers who have at least one child under- five years of age with diarrhea needing home management. The study was conducted in three phases. Phase one was a base line survey for the mothers. Intervention phase including different health education approaches, home visits, group sessions and distribution of mother cards through community volunteers and researchers. Post intervention survey using the same pre-intervention questionnaire, and observation of mothers managing their children.

**Results:**

Results showed that knowledge of mothers about definition of diarrhea, its danger, when to seek medical help and the three rules of home management which was found to be 35, 28, 13 and 29% improved significantly after intervention to 91, 94, 92 and 93% respectively with a very high significant level.

**Recommendations:**

We recommended that volunteers are effective health education provider especially on household based intervention. Health services should support the community based interventions to reinforce the knowledge and practices of mother towards the sick children.

## INTRODUCTION

Knowledge, attitudes, and practice (KAP) studies that evaluate the knowledge of communities regarding disease uncovered marked deficiencies in the level of education given to people at risk of contracting disease. Therefore, health education programmes aimed at improving community participation in control strategies were recommended. A control programme could be achieved successfully if implemented by the village head. The information gathered through these studies could serve as a basis for a quality improvement programme by educating health professionals and the community on disease and its management, to ensure the availability of medications in health facilities, and improve patients’ access to health services.[1]

Diarrhea is defined as the passage of three or more loose or watery stools (loose stools take the shape of the container) in a 24-hour period.[2] Current estimates of the global burden of diarrheal disease were compared with previous estimates using data collected in 1954 to 1979 and 1980 to 1989. A structured literature review was used to identify studies on morbidity rates by prospective surveillance of stable populations and studies that dealt with mortality attributable to diarrhea by means of active surveillance. For children less than five years of age in developing areas and countries, there was a median of 3.2 episodes of diarrhea per child-year. This indicated little change from incidence previously described. Estimates of mortality revealed that 4.9 children per 1000 per year in these areas and countries died as a result of diarrheal illness in the first five years of life, a decline from the previous estimates of 13.6 and 5.6 per 1000 per year. The decrease was most pronounced in children less than one year old. Despite the improving trends in mortality rates, diarrhea accounted for a median of 21% of all deaths of children aged less than five years in these areas and countries, with 2.5 million deaths per year. There has not been a corresponding decrease in morbidity rates attributable to diarrhea. As population growth is focused in the poorest areas, the total morbidity component of the disease burden is greater than that obtained previously.[3,4]

Health education is the first component in primary care. It is defined as ‘any combination of learning experiences designed to lead to a situation where people know how to attain health, do what they can individually and collectively to maintain health and seek help when needed.’ Health education aims at changing of behavior and life style.[5]

The importance of home management of diarrhea lies in the fact that diarrhea starts at home, and continues at home on return from being seen at a health facility. A lot of fluid is lost in diarrheal stools, and if suitable fluids are given in adequate volumes soon after the onset of diarrhea, dehydration can often be prevented. However, the fluids given must meet certain criteria when given in large volumes. They are easy to prepare, but should be familiar and acceptable to the child, and effective. Specific recommendations such as rice water, soup, yogurt, and oral rehydration salt (ORS) agreed on by the UNICEF are important.

Feeding of the child should continue, particularly with plenty of nutritious food, to prevent any decline in growth necessary during and after the episode of diarrhea. Mothers usually prefer traditional methods of managing diarrhea, and only seek medical advice when it fails. Unfortunately, this is usually too late. The child is either already dehydrated or has started to lose weight. Therefore, it is important to notice and identify certain symptoms or signs in order to seek medical advice promptly.

Previous studies done in Sudanese villages around Khartoum city showed evidence of the significant role of health education in improving the management of diarrhea at home.[6–8]

The efficacy of health education in improving home management of diarrhea has been proved and recommended in some studies in sub-Saharan African countries,[9,10] India, and Nepal.[11,12,13]

This paper has three objectives; first, to assess KAP of mothers on home management of diarrhea; second, to evaluate the effect of educating mothers on health matters to improve health care for children less than five years of age with diarrhea; finally, to improve the competence of mothers in managing diarrhea at home.

## MATERIALS AND METHODS

This is a quasi-experimental study held in Al Maki neighborhood which is located in Greater Wad Medani locality, Gezira State, Central Sudan. The total population of Almaki neighborhood is 11624 of different ethnic backgrounds. The total number of families is 1183. The target group of this study was mothers who had at least one child less than five years of age with diarrhea needing home management. The World Health Organization (WHO) definition of diarrhea which is the passage of three or more loose or watery stools (loose stool being stools that would take the shape of the container) in a 24-hour period was used for the purpose.[2] The degree of dehydration was also determined according to the criteria set by WHO. There is no dehydration in a child with diarrhea who shows only one or none of the four cardinal features of dehydration (general condition, sunken eyes, degree of thirst, and degree of loss of skin elasticity). The mothers were selected using systematic random sampling technique. A total of 118 mothers were selected, giving almost 10% of the total number of families, eight of whom dropped out as a result of poor compliance. Verbal consent was taken from mothers before their enrolment in the study.

Health education was provided by 24 female volunteers, who were members of the Red Crescent, with secondary level of education, selected from the same area. They had previous experience in voluntary work in national immunization drives, a week for the control of diarrhea. The health education sessions were supervised by eight nutritional educators, vaccinators, and teachers from Almaki area. The work was implemented in three phases. In phase one, a baseline survey was conducted with the aim of measuring the KAP of the mothers in managing diarrhea at home. The training of the volunteers took place during this phase. A health education package on the home management of diarrhea was formulated and tested. This dealt with the definition, etiology, assessment of complications, and management of diarrhea.

This curriculum was taught in a three-day course of 14 contact hours. The methods used in the training were lectures, group discussion, a video show, role plays, and clinical sessions using mother cards. Participants were evaluated by means of multiple choice questions, group work presentation, and interviews by supervisors. Twelve sessions of focus group discussion were held with mothers and supervisors to determine the implementation of the health education sessions.

In phase two, the mothers were divided into 24 groups, five households in each group supervised by one volunteer, and one supervisor having the oversight of three volunteers. Volunteers did one session per week for their groups for four months, meeting in a different home for each session. The supervisor made two visits each to her designated household, and the researcher held a session once every month at the health centre for the mothers, volunteers, and supervisors to consolidate the issues raised in the health education, followed by a meeting to discuss the different reports presented by the volunteers on the progress of work, and resolve any issues or problems.

In phase three, the women had no instructions for three months and then were given the postintervention interview. The postintervention survey was conducted using the same preintervention questionnaire, and the mothers’ reports on the preparation of the ORS and the quantity administered.

The Statistical Package for Social Sciences (SPSS) software programme was used for data analysis. Frequency distributions were obtained and descriptive statistics was calculated. Also, the Chi-square test was used to test the differences between the pre- and postintervention situation.

## RESULTS

Table 1 shows that the majority of the mothers were young, in the 20 to 29 years age group, constituting 51.8% of the women, followed by women between 30 and 39 years of age constituting 38.2%. Only 10% were between 40 and 49 years of age. The same Table shows the level of education of the mothers as follows: 19.1% were illiterate, 42.8% had intermediate schooling, 21.8% had secondary education, and 25.5% were university graduates.

The majority of fathers (70.9%) were unskilled laborers, 16.4% were workers, and 12.7% were employees. Families with one child less than five years were 45.5% with two children were 37.3%, three children were 11.8%, and only 5.5% had four children.

Table 1 also demonstrates children’s age. Children less than one year of age were 33.6%, two years were 30%, three years were 24.5%, and four-year-old children were 9.1%.

**Table 1:** Background characteristics of the women

Characteristics	N	%
Age group of the mothers		
20−29	57	51.8
30−39	42	38.2
40−49	11	10
Mother’s educational status		
Illiterate	21	19.1
Primary	19	17.3
Intermediate	28	25.5
Secondary	22	20.0
University	20	18.2
Father’s occupation		
Employee	14	12.7
Worker	17	16.4
Unskilled jobs	78	70.9
Number of children under five years in families		
One child	50	45.5
Two children	41	37.3
Three children	13	11.8
Four children	6	5.5
Age of the last child		
0−12	37	33.6
13−24	33	30
25−36	27	24.5
37−48	10	9.1
49−60	3	2.7
Total	110	100

One important determinant affecting the standard of living and social welfare was family income. Table 2 shows the distribution of sampled mothers by monthly income. It is clear that the majority of the families had no regular income, 3.6% of the families had an income of less than 100 SDG, while only 14.5% of them had an income of more than 100 SDG per month.

**Table 2:** Family income, presence of animals and latrines in the house

Characteristics	N	%
Family income per month (SDG)		
Less than 100	37	33.6
More than 100	16	14.5
Not constant	57	51.8
Presence of animals in the house		
Yes	19	17.3
No	91	82.7
Presence of latrines in the house		
Yes	105	95.4
No	5	4.6
Total	110	100

The children’s environment had a major influence in their well being and survival. Table 2 indicates that 82.7% of the families had no animals in their homes. Only 17.3% reported the presence of animals in the house. On sanitation, 95.4% of the families had latrines, compared with only 4.6% who had no latrines in their houses.

**Figure 1:**
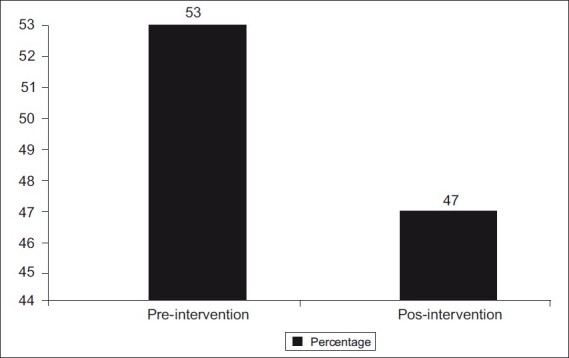
Prevalence of diarrhea in the last two weeks

Figure 1 shows the prevalence of diarrhea during the two weeks before the survey, pre- and postintervention. The figures show that the prevalence of diarrhea dropped from 53% preintervention to 47% postintervention.

Table 3 shows the mothers’ knowledge of the definition of diarrhea, its cause, seriousness, complications, prevention, when and where to seek medical help and knowledge about three rules of home management. The table shows that all those indicators improved significantly as a result of the intervention (Chi square values are significant for all indicators).

**Table 3:** Mothers knowledge in relation to definition of diarrhea and home management

Indicator	Before			After			Chi Square	*P* value
	Know No (%)	Partially know No (%)	Do not know No (%)	Know No (%)	Partially know No (%)	Do not know No (%)		
Mothers knowledge about definition of diarrhea	35(35)	53 (53)	22 (22)	91 (91)	10 (10)	9 (9)	11.7	0.020*
Mothers knowledge about danger of diarrhea	28 (28)	47 (47)	35 (35)	94 (94)	8 (8)	8 (8)	19.94	0.001**
Mothers knowledge on when to seek medical advice	13 (13)	79 (79)	18 (18)	92 (92)	8 (8)	10 (10)	4.7	0.031*
Mothers knowledge of the three rules of home case management	29 (29)	46 (46)	35 (35)	93 (93)	7 (7)	10 (10)	11.71	0.020*

* Significant at 5% level

** Significant at 1% level

Table 3 shows that knowledge of mothers of the definition of diarrhea, its dangers, when to seek medical help, and the three rules of home management which were 35, 28, 13, and 29%, improved significantly after intervention to 91, 94, 92, and 93%.

**Figure 2:**
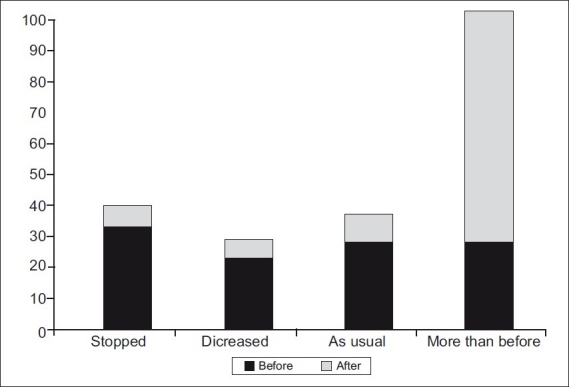
Amount of food given to child with D D Chi Square = 28.94; *P* value = 0.021

Figure 2 shows significant improvement of feeding practices during a diarrheal episode. The percentage of mothers who stopped feeding dropped from 30 to 7%, whereas those who gave more food increased from 25 to 77%.

**Figure 3:**
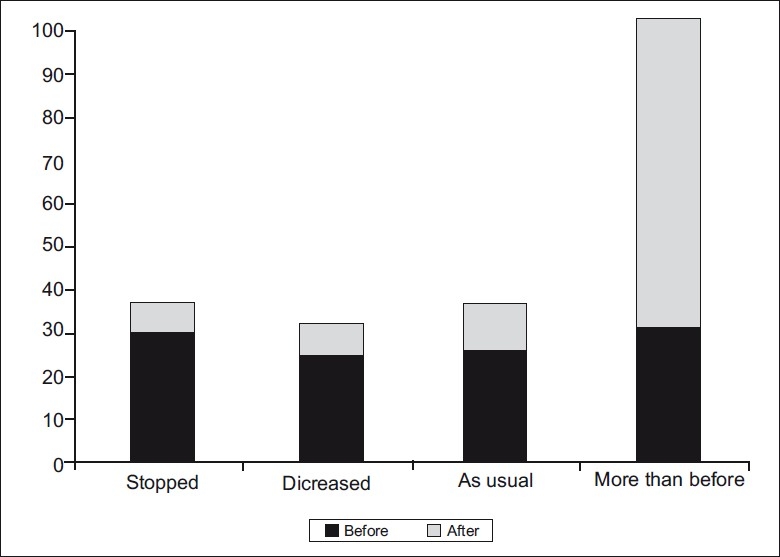
Amount of fluid given to child with D D Chi Square = 30.2; *P* value = 0.017

Figure 3 shows that 49% of the mothers either stopped or reduced the amount of fluid given to the child with diarrhea, but 23% of them did not change the amount of food given to the child. After intervention, this decreased to 15 and 11%, respectively, whereas those who gave more fluid increased from 28 to 74%. Table 4 shows that the mothers’ knowledge of regular weighing of their children improved significantly from 25.5 to 86.3%.

**Table 4:** Mother’s knowledge about the weight monitoring of her child

Mothers knowledge about the weight of a child	Before		After		*P* value
	Frequency	%	Frequency	%	
Know	28	25.5	84	86.3	0.025*
Do not know	82	74.5	26	63.6	
Total	110	100	110	100	

* Significant at 5% level

## DISCUSSION

According to the multiple indicators survey which was conducted in Central Sudan in 2000, the prevalence rate of diarrhea in children under the age of five years was 27%, while for children less than two years of age it was 39.2%.[14]

The household survey conducted in northern Sudan to assess mothers’ KAP toward home management of a child with diarrhea showed that the knowledge of mothers of the three rules of management (increase in fluids, continuation of feeding, and getting medical care) showed that 15% of the mothers surveyed knew these three rules.[15] The 35% found at the beginning of our study improved dramatically after intervention to 93%. These results were consistent with other studies done in Sudan.[6–8]

The study shows that the significant improvement in the knowledge of the mothers of the three rules of home management after intervention was consistent with the findings of other studies, proving that health education using volunteers is an effective means of improving mothers’ competence in caring for their children at home during episodes of diarrhea. The level of education of the mothers was low, some were illiterate. However, most mothers had two or more children, which indicated that they were only housewives, this means more involvement of mothers in home business, reaching mother at home with somebody who is familiar to them, will let them don’t feel shy to ask about unclear points, all these are assets in receiving the health education messages.

Weighing of children is an important means of monitoring the growth of children under the age of five years, and should be done simply by plotting the weight at definite intervals and calculating the rate over specified periods as part of the welfare of children under five years old.[15] To achieve this in Sudan, a mother’s card was designed, but the knowledge of the mothers of its importance was very poor in our study. Their knowledge of monitoring their children’s weight was significantly improved after intervention. This is particularly important, knowing that diarrhea is the most important cause of malnutrition in the world, because approximately 2% of the child’s body weight is lost each day when a child has acute diarrhea.[16]

## CONCLUSION AND RECOMMENDATION

We conclude that health education activities like personal communication, focused group discussion, lectures, and home visits achieved through volunteers produced a significant improvement in mother’s KAP with respect to home care for children under the age of five with diarrheal disease. Finally, we recommend that different approaches used in health education would be an effective strategy for the improvement of mothers’ competence in managing their children at home. Health services should support the community-based interventions to reinforce the activities, raise the referral level, and improve consultation.
